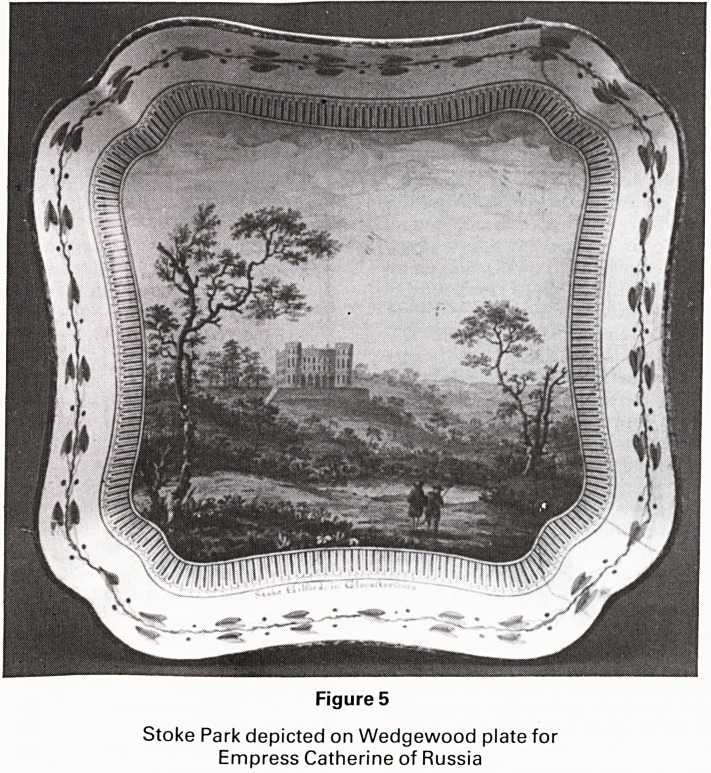# The History of Mental Handicap in Bristol and Bath (Part 1)

**Published:** 1986-06

**Authors:** J. Jancar

**Affiliations:** Consultant Psychiatrist, Stoke Park Group of Hospitals and Clinical Lecturer in Mental Health (Mental Handicap) University of Bristol (Presidential Address, Bristol Medico-Chirurgical Society)


### The History of Mental Handicap in Bristol and Bath (Part I)

**J. Jancar M.B., B.Ch., B.A.O., F.R.C.Psych., D.P.M.**
Consultant Psychiatrist, Stoke Park Group of Hospitals
and
Clinical Lecturer in Mental Health (Mental Handicap) University of Bristol
(Presidential Address, Bristol Medico-Chirurgical Society)

The cities of Bristol and Bath have played an eminent role in the history of Mental Handicap, but time will not permit me to deal with this in detail. I shall try to concentrate on the most important people and places, and others I will mention in passing. Unfortunately, documentation is rather scanty particularly on the pre and post Reformation era. We are very fortunate that we have more detailed documentation on the Holy Cross Hospital in Bath, which appears to be the oldest Mental Handicap Hospital in Great Britain.

### Holy Cross Hospital, Bath

I feel that the Romans contributed to the foundations of this hospital when they built Fossway Road on the outskirts of Bath which the pilgrims later used to visit Glastonbury. Holy Cross Hospital (Figure 1) was nearly destroyed during the last war. A bomb fell not far from the hospital and partially damaged the nearby Chapel, which is now nicely rebuilt and redecorated.

Sited next to the Holy Cross Hospital is the Chapel of St Mary Magdalen, Holloway (Holy Way) (Figure 2). The earliest edifice on this site would appear to have been erected before the Norman Conquest, as stated at the end of an old Saxon Manuscript of the Four Gospels in Latin dated 983 which is preseved in the library of Corpus Christi College, Cambridge. The Chapel was built beside a spring water well where the pilgrims used to stop to rest and pray. A hostel was built next to the Chapel in 1212 and in 1235 it was labelled as a lepers house. In 1263 an undated deed appeared in the Bath Cartulary which records a grant of land by John Wyssey to the Hospital of St Mary Magdalen in Holeweia and to the brothers and sisters there serving God. At the dissolution of the monasteries in 1534, the Magdalen Chapel remained open and the Holy Cross Hospital continued with the reception of lunatics (Shuttleworth and Potts, 1910). Benedictine monks from Bath Abbey had a special Festival of Rudmas Day—3 May—the feast of the invention of the Holy Cross for even as late as 1794, a fair was held in the vicinity of the hospital on this date. In the present Verger's house, former hospital, is a tablet which reads in Latin: (Figure 3)

'Anno salutis MDCCLXI haud multo post Inaugorationem Georgii Tertii auspicatifsimi Regis: Hoc MOROTROPHIUM quod ante ducentos et septuaginta annos JOHANNES CANTLOW prior Bathoniensis fundavit Vetustate pene collapsum ieaedificavit DUEL TAYLOR Bathiniae Rector et hujus Hospitii Magister'.

**Figure 1**

Holy Cross Hospital today

**Figure 2**

Holy Cross Hospital and Chapel of St Mary Magdalene early 19th Century

**Figure 3**

Tablet in the former Holy Cross Hospital

The sense of this inscription was translated by the British Museum:

'In the year of Our Lord 1761, not long after the coronation of the most illustrious King George III, this lunatic asylum, founded two hundred and seventy years before by John Cantlow, Prior of Bath, and almost collapsed through age, was rebuilt by Duel Taylor, Rector of Bath, and master of this lodging'. (1491 is therefore the year of the foundation of the hospital).

The word MOROTROPHIUM on the tablet is of great interest. We were not happy with the translation. After a long search and with the help of the Bristol Medical Library, we were able to get the correct translation: MOROTROPHIUM consists of two Greek words, MOROS—stupid/feeble minded, moron—and TROPHEIA, which became latinised to TROPHIUM—place for nursing or caring, therefore the modern translation should read 'Mental Handicap Hospital'. This is confirmed by the last but one word on the tablet 'Hospitii' meaning home for the needy or afflicted.

### St Peter's Hospital, Bristol

St Peter's Hospital in Bristol deserves to be much better known than it is, having been probably the earliest asylum of any size in the country apart from Bethlem. In 1698 the first patients were received. The building was of great antiquity, having been in use as a private residence since 1402, or possibly even earlier. During most of the seventeenth century it was used partly as a residence and partly as a sugar refinery. It is referred to as the 'Sugar House'.

From 1696 to 1698 it was in use as a mint, and continued to be popularly known as 'the Mint'. Patients here were admitted under the Bristol Poor Act of 1696 which was an historic piece of local legislation, anticipating by 86 years one of the provisions of Gilbert's Act of 1782, whereby parishes were permitted to combine for the more efficient administration of the Poor Law. It was inspired and published by John Cary in 1695. The first inmates were 100 boys, but these were soon joined by other inhabitants, some aged, some infirm, some lunatic and some Mentally Handicapped. Thus the transformation of the workhouse into a hospital began almost at once. St Peter's Hospital continued to be used as a lunatic asylum until 1861 when its patients were transferred to a newly opened lunatic asylum at Fishponds. The destruction of St Peter's Hospital by enemy fire in 1940 was a tragic loss to the City of Bristol. Dr. T. Dover was the first consultant physician at St Peter's Hospital. He is remembered for his 'Dover's powder' (pulvis ipecacuanhae et opii).

In 1735 the Bristol Royal Infirmary was instituted where psychiatric patients were seen in the outpatients' department by Psychiatrists including Dr. E. Long Fox and Dr. J. C. Pritchard who wrote one of the first text books on insanity. I will mention a few more asylums in Bristol as there was no clear distinction between Mental Illness and Mental Handicap at this stage. Very often Mentally Handicapped people were admitted to a lunatic asylum. In 1740, a private lunatic asylum was opened in Fishponds by Dr. J. Mason who earlier managed his father's small mental establishment at Wickwar. About 1790, the pioneering treatment of the Mentally Ill by 'moral' treatment and group therapy was first introduced by Dr. E. L. Fox in a small Quaker's asylum at Cleeve Hill, Downend, then in Brislington House (1804) and later Northwood Asylum in Winterbourne which was opened by Henry Fox in 1845.

At the time of the cholera epidemic in Bristol in 1832, some patients were transferred from St Peter's Hospital to an old prison building at Stapleton, which was built in 1779 to house Spanish, Dutch and French naval prisoners of war. Most of the institution was rebuilt in 1861 and renamed Stapleton Institution for Mental Defectives in 1918. In 1948 it became Stapleton Hospital for Geriatric Illness and was renamed again in 1956 as Manor Park Hospital.

### Cambridge House

Cambridge House, Flax Bourton was opened in 1842 as a Poor Law Institution. In 1917, part of Cambridge House was used to house the Mentally Handicapped and later became known as Farleigh Hospital and in 1971 joined the United Bristol Hospitals with Yatton Hall which opened in 1919. Both hospitals are now providing the services for the Bristol and Weston Health Authority.

In 1846 two sisters by the name of White opened a small school for Mentally Handicapped children in Bath and this eventually developed into the Rockhall House School, opened in 1891 and was amalgamated later with the Holy Cross Hospital mentioned previously.

Mary Carpenter, a Victorian reformer, opened Red Lodge Reformatory School in 1854 and the Kingswood Approved School in 1852. She was a pioneer in the treatment of juvenile delinquency.

In 1890, Miss Harriett Wemyss opened St. Mary's Home, Painswick, near Stroud for Mentally Handicapped girls. This home is still housing Mentally Handicapped people from the Gloucester Health Authority.

Three years later a similar home for Mentally Handicapped girls was opened in Bristol; the Chasefield Home, 188 Fishponds Road. It was described as being in the country and yet within easy reach of Bristol.

Towards the end of the last century and at the turn of this century there were four remarkable people who played a very important part in the development of the services for the care of Mentally Handicapped people in Bristol, Bath and Somerset. A great pioneer in this field was Miss Norah Fry. The Norah Fry Hospital was named after her in her maiden name, and later when she married, the first Chair in Mental Health at Bristol University was also named after her; Mrs Norah Cook Hurle. Therefore, it is not surprising that the present holder of the Chair, Professor G. Morgan is very much involved in the education of undergraduates and postgraduates in Mental Handicap. Another influential man was the late Marquess of Bath and the two other people were the Reverend Burden and Mrs Burden.

### The Burdens

The Reverend Harold Nelson Burden (Figure 4) and Mrs Katherine Mary Burden came to Bristol in 1895, when Mr Burden was appointed chaplain to Horfield Prison. Their arrival in Bristol opened a new chapter in the history of the care and treatment of down and outs and especially of Mentally Handicapped people. Mr and Mrs Burden first came in contact with the people in real need and misery when they worked in the East End of London. They were also influenced by the work of Miss Octavia Hill (1842–1912), who was regarded as an authority on the lives of the poor in London and was a friend and disciple of Ruskin.

Mrs Burden worked for some time as his assistant. Mr and Mrs Burden were instrumental in the promotion and erection of the Royal Victoria Home near Horfield Prison.

The passing of the Inebriates Act of 1898 made much larger accommodation necessary for inebriates. Premises were therefore taken at Brentry and the assistance of County and County Borough Councils was sought. Twenty-four of these decided to contribute to the establishment of the Brentry Certified Inebriate Reformatory. Two villages were erected—the upper for females and the lower for males. Brentry House was adapted for offices and a residence for a superintendent. For three years, both Mr and Mrs Burden gave it their continuous attention. Every women who entered the institution came under Mrs Burden's influence and received her help. Mr Burden remained on the Brentry Certified Institution Board of Management as vice-chairman until his death in 1930.

**Figure 4**

The Reverend Harold Burden

As the Inebriates Act proved to be ineffective and as the number of Mentally Handicapped patients was increasing, the Brentry Certified Inebriate Reformatory ceased to exist on the 3rd January 1922 when it became Brentry Certified Institution within the meaning of the Mental Deficiency Acts, 1913 and 1919. The newly designated institution provided 220 beds for male Mentally Handicapped patients over the age of 18 years. The aim of the institution was to occupy the patients as much as possible.

In 1930 Brentry Certified Institution was renamed Brentry Colony and Dr. R. G. Rudolf was appointed Medical Superintendent. He was a renowned researcher and produced a number of very interesting publications. In 1948 Brentry Colony merged with Hortham Colony. (Jancar, 1972).

### Stoke Park Hospital

Mr and Mrs Burden first bought Eastern Counties Institution, East Harling, Norfolk in 1904 and then Sandwell Hall, Handsworth, Staffordshire, in 1906. In 1909 they acquired from the Duke of Beaufort, the Dower House, Stoke Park, later known as Stoke Park Colony. Whittington Hall, Chesterfield was opened in 1912.

Stoke Park Colony was opened on 1 April 1909. The Dower House, which was the focal point of the future hospital has an interesting history. The first mention of Stoke in historical records is the Domesday Book. After the Norman invasion of 1066, the Saxon Duns was dispossessed and the Manor of Stoche was given by William the Conqueror to one of his lieutenants, Osborne Giffard, who came from Scie in Normandy. His family had been known as the Lords of Longueville-la-Giffard. After the death of John, the last Giffard, the Manor of Stoke Gifford passed into the hands of Maurice de Berkeley, who became the founder of the Stoke Gifford branch of the family. The Manor of Stoke Gifford (*Dower House of today*) was rebuilt in 1760–1764, with the motto 'Mihi Vobisque' (Mine and Yours), by Norborne Berkeley who was in 1768 appointed the Governor of Virginia. He died in the USA in 1770. The Manor of Stoke Gifford passed through his sister Elizabeth, who was by then the Duchess Dowager of Beaufort, to the Beauforts and was for a long time used as a Dower House to Badminton. The 10th Duke of Beaufort sold the Manor in 1907 to the Reverend Burden.

Stoke Gifford was depicted in 1774 by an artist who painted the English scene for the Wedgwood Pottery, for an order of 952 pieces of dinner and dessert service for Empress Catherine II of Russia.

This service is now on display at the Winter Palace (Hermitage) in Leningrad. (Fig 5) Bristol pottery and local artists frequently painted Stoke Park.

There are two other interesting features which I would like to mention. The first is the bandstand which was sited in the woodland near to the Dower House. Unfortunately the only remaining evidence of this band stand in the overgrown woodland is the access to it and the underpass, both are still traceable. Another interesting feature is the tomb of a horse called Matilda which won the St Ledger in 1827. The picture of this horse is in Badminton House.

The Reverend Burden travelled through Europe trying to find the latest treatment for Mental Handicap and he brought back the idea for Heliotherapy, an Open-air treatment practised on the continent at that time, to increase vitality, weight and resistance to disease. He built five two bedded revolving houses among the trees near the

*(continued on page 63)*

606 REM * change disc *

I doubt if any Dutchman, or even solemn Chinese mandarins at breakfast would turn a hair. I certainly shall not, but then that is not really talking.

607 EXIT.

J. D. D.

### REFERENCES

1. WILLIAMSON, H. R. (1947) Chinese: basic Mandarin. McKay Publishing, Inc., New York.
2. ILLINGWORTH, V., GLASER, E. L. and PYLE, I. C. (1983) Dictionary of computing. Oxford University Press, Oxford.

### The History of Mental Handicap *(continued from page 55)*

**Figure 5**

Stoke Park depicted on Wedgewood plate for Empress Catherine of Russia hospital ward for convalescent patients and a sheltered enclosure between the wards used for sun baths. During the summer, large tents were provided as domitories, dining rooms and play rooms. As the demand for more beds grew, Mr Burden purchased more property, and Heath House was acquired in 1911 and Stapleton Grove-Beech House (now Purdown Hospital) in 1916 followed by Hanham Hall and Leigh Court in 1917.

*To be continued in the next issue.*

## Figures and Tables

**Figure 1 f1:**
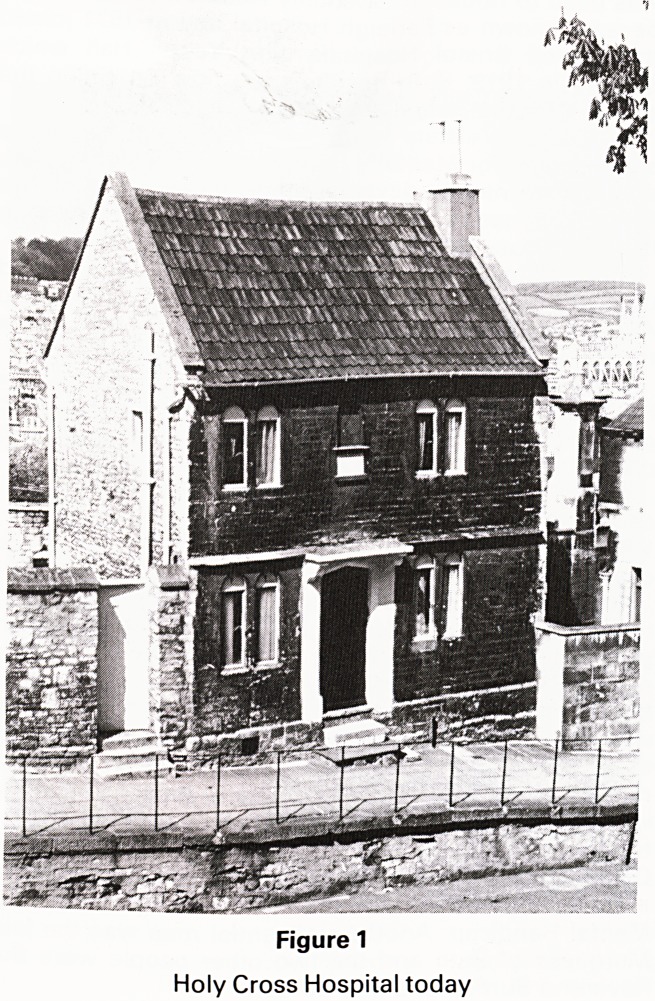


**Figure 2 f2:**
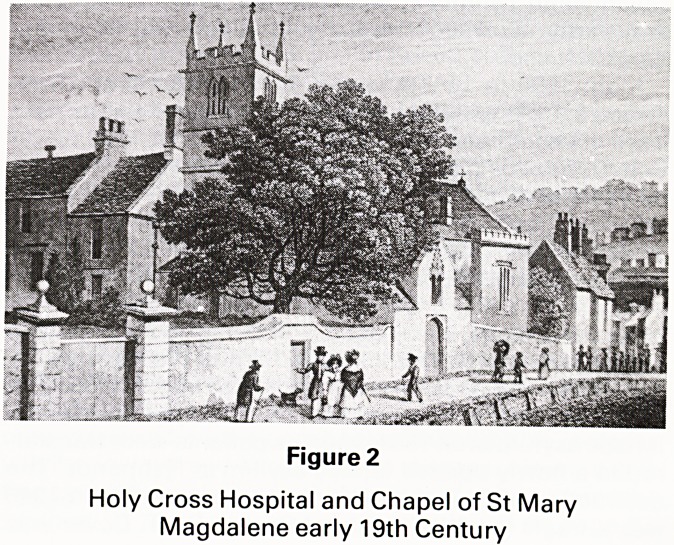


**Figure 3 f3:**
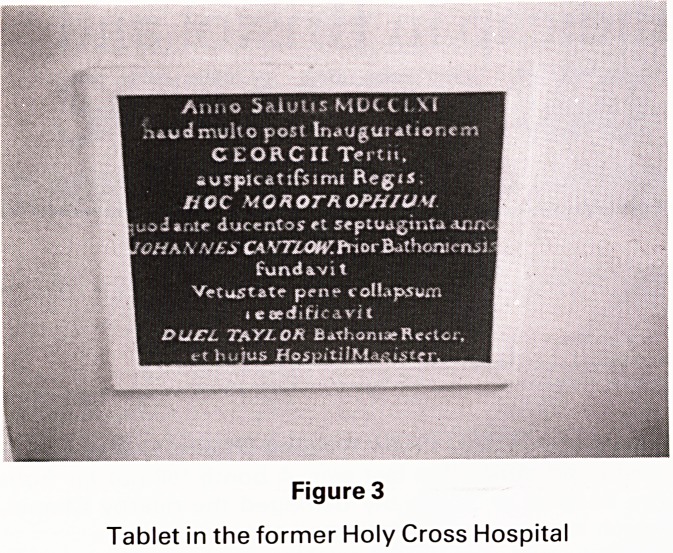


**Figure 4 f4:**
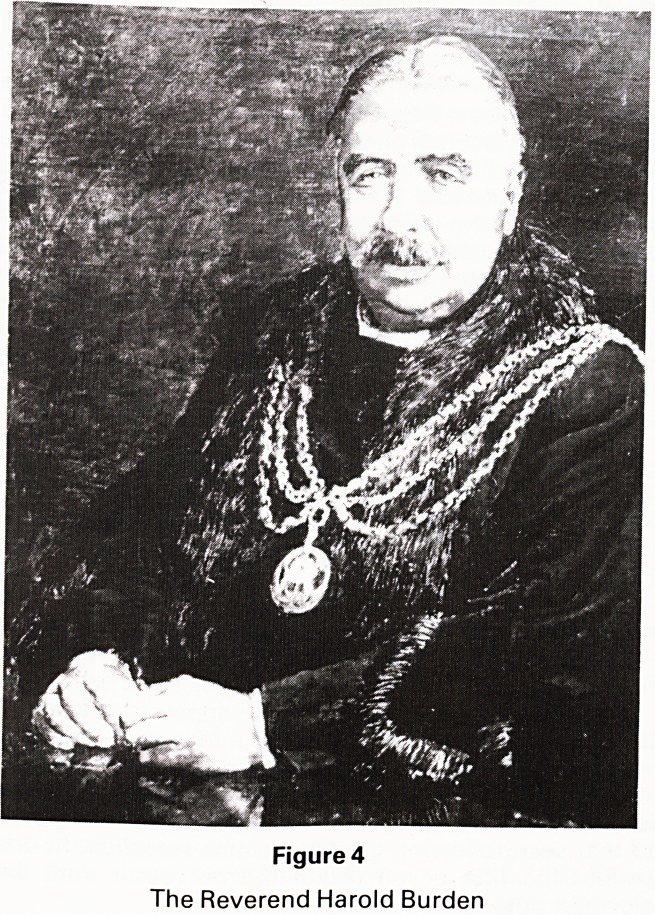


**Figure 5 f5:**